# Antibiotics Effects on the Fecal Metabolome in Preterm Infants

**DOI:** 10.3390/metabo10080331

**Published:** 2020-08-13

**Authors:** Laura Patton, Nan Li, Timothy J. Garrett, J. Lauren Ruoss, Jordan T. Russell, Diomel de la Cruz, Catalina Bazacliu, Richard A. Polin, Eric W. Triplett, Josef Neu

**Affiliations:** 1 Division of Neonatology, Department of Pediatrics, University of Florida, Gainesville, FL 32610-0296, USA; laura.patton@peds.ufl.edu (L.P.); linan@peds.ufl.edu (N.L.); lruoss@peds.ufl.edu (J.L.R.); ddelacruz@ufl.edu (D.d.l.C.); cbazacliu@peds.ufl.edu (C.B.); 2 Department of Pathology, Immunology and Laboratory Medicine, College of Medicine, University of Florida, Gainesville, FL 32610, USA; tgarrett@ufl.edu; 3 Department of Microbiology and Cell Science, Institute of Food and Agricultural Sciences, University of Florida, Gainesville, FL 32603, USA; russell.j.7@ufl.edu (J.T.R.); ewt@ufl.edu (E.W.T.); 4 Department of Pediatrics, College of Physicians and Surgeons, Columbia University, New York, NY 10032, USA; rap32@cumc.columbia.edu

**Keywords:** antibiotics, metabolome, preterm infants

## Abstract

Within a randomized prospective pilot study of preterm infants born at less than 33 weeks’ gestation, weekly fecal samples from 19 infants were collected and metabolomic analysis was performed. The objective was to evaluate for differences in fecal metabolites in infants exposed to antibiotics vs. not exposed to antibiotics in the first 48 h after birth. Metabolomics analysis was performed on 123 stool samples. Significant differences were seen in the antibiotics vs. no antibiotics groups, including pathways related to vitamin biosynthesis, bile acids, amino acid metabolism, and neurotransmitters. Early antibiotic exposure in preterm infants may alter metabolites in the intestinal tract of preterm infants. Broader multi-omic studies that address mechanisms will guide more prudent antibiotic use in this population.

## 1. Introduction

Antibiotics (abx) are the most common drugs prescribed to infants in the neonatal intensive care unit (NICU), largely because of the concern for sepsis [1,2,3]. The dogma for most neonatologists is that providing pre-emptive routine antibiotics for very-low-birthweight infants shortly after birth saves lives without attendant complications. Current evidence to support the practice of routinely administering antibiotics in preterm neonates is inadequate. Despite recent initiatives to limit antibiotic use and the low incidence of early onset sepsis (EOS), most infants born at less than 33 weeks’ gestational age are exposed to a course of antibiotics shortly after birth. Recent trials suggest that use of antibiotics in preterm infants have adverse consequences such as an increase in necrotizing enterocolitis, chronic lung disease and death [4,5,6]. Understanding the long-term effects of antibiotics on preterm infants may further guide this practice.

It is well known that intestinal microbial metabolism plays a major role in metabolite production [7]. Metabolic alterations during a critical window of development, such as the fetal or immediate neonatal period, are known to elicit life altering effects [8,9]. Previous studies in adult mice show that antibiotic treatment disrupts intestinal homeostasis and has a profound impact on the intestinal metabolome [10]. Many adult human microbiome studies conclude that significant metabolic perturbations are secondary to antibiotic administration in relation to *Clostridium difficile* diarrheal infections and antibiotic associated diarrhea [11,12]. Specific effects of antibiotics on the fecal metabolome have been studied in murine models [10,13,14]. Of particular interest, gentamicin and ceftriaxone produced increased levels of secondary bile acids and oligosaccharides but decreased levels of primary bile acids, branched-chain amino acids, and aromatic amino acids [15]. Antibiotics affect most metabolites that are detectable in the human intestinal tract, including ones critical for host physiology. Data on the effects of antibiotic use on the intestinal metabolome in preterm infants remains sparse, with few observational or retrospective studies available [16,17,18].

Our group designed a pragmatic randomized controlled pilot study to evaluate safety and feasibility of not giving routine antibiotics [19] to symptomatic preterm infants shortly after birth. (Routine Early Antibiotic use in SymptOmatic preterm Neonates—“REASON”). This trial was also designed to evaluate the fecal microbiome and metabolome longitudinally in these infants. The REASON trial randomized preterm infants with symptoms of prematurity to receive (routine practice) vs. not receive antibiotics shortly after birth. Weekly stool samples and extensive clinical meta-data were collected longitudinally during the hospitalization. Preliminary results showed that antibiotic use alters the microbiome and metabolome, including metabolites related to the gut-brain axis [20]. Of particular interest, gamma aminobutanoate (GABA), which is a major neurotransmitter, was found to be affected by antibiotic use. In the metabolomics sub analysis within the REASON trial, relative abundance of *Veillonella* and *Bifidobacterium* in the stool were both significantly affected by the administration of antibiotics. *Veillonella* showed a positive association with GABA, suggesting an antibiotic-mediated perturbation of a metabolite known to affect the gut-brain axis [20]. These variances could be significant for the lifetime health of the individual since they are occurring during a very sensitive window for development [9].

Administration of various types of antibiotics has also been shown in multiple animal models to produce significant changes on both the microbiome and metabolome [21,22]. One neonatal model theorized a personalized metabolome and microbiome based on antibiotic exposure [18]. Here we describe the differences in fecal metabolomic profiles of preterm infants who were randomized to receive vs. not receive antibiotics shortly after birth.

## 2. Results

### 2.1. Study Population

Ninety-eight neonates, born at gestational ages of less than 33 weeks, were placed into three groups (A, C1-abx, C2-no abx). Nineteen of these neonates underwent metabolomics profiling in this study; six in group A, eight in group C1-abx, and five in group C2-no abx. Infants with symptoms not expected for gestation or infants at high risk for infection (i.e., group B Streptococcus positive mother without adequate prophylaxis or chorioamnionitis) were placed into group A. Group C consisted of infants with symptoms expected for prematurity such as respiratory distress, and without significant risk factors for infection. Infants in group C were randomized to receive (C1-abx) or not receive (C2-no abx) antibiotics as described in Ruoss et al. [7,19]. Three infants from this subset were randomized to C2 (no abx) and changed to receive antibiotics within 48 h after birth, and therefore are included in the C1-abx group for this analysis. A total of 123 stools samples were collected: 43 for group A, 54 for group C1-abx, and 26 for group C2-no abx (Table 1).

### 2.2. Metabolomics-Comparison of Three Groups

**Figure 1:**
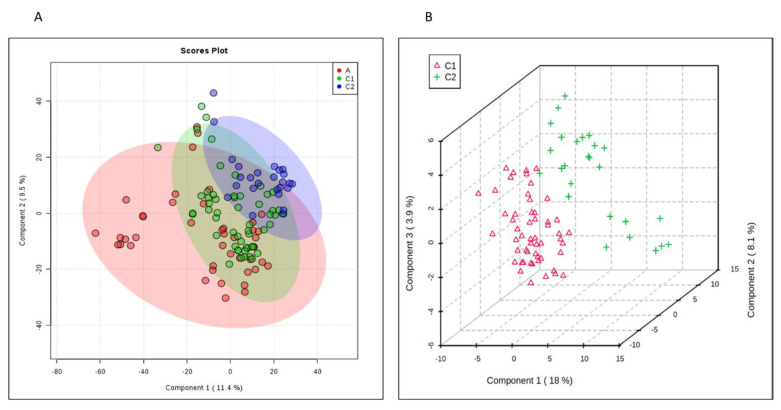
Comparison of Groups A, C1 and C2. (**A**) PLS–DA plot depicting overlapping components of groups A (red), C1 (green) and C2 (blue). (**B**) 3D plot showing further delineation of groups C1 (red) and C2 (green).

PLS–DA (partial least squares discriminant analysis) cluster analysis of the metabolites (Figure 1A) depicts variances in the metabolic profiles between the three groups (Q2 = 0.49). The groups receiving antibiotics (A and C1–abx) notably have more overlap in their metabolic profile. The no antibiotic group (C2–no abx) clusters further apart with some overlap. 3D analysis of the randomized group C1 and C2 (Figure 1B) better illustrates the PLS–DA separation of their metabolites. While the Q2 value is above 0 and demonstrates predictive prevalence, the low value suggests there is more variability within the profiles, and that more information would result in a higher Q2 value and would indicate a more accurate model.

**Figure 2:**
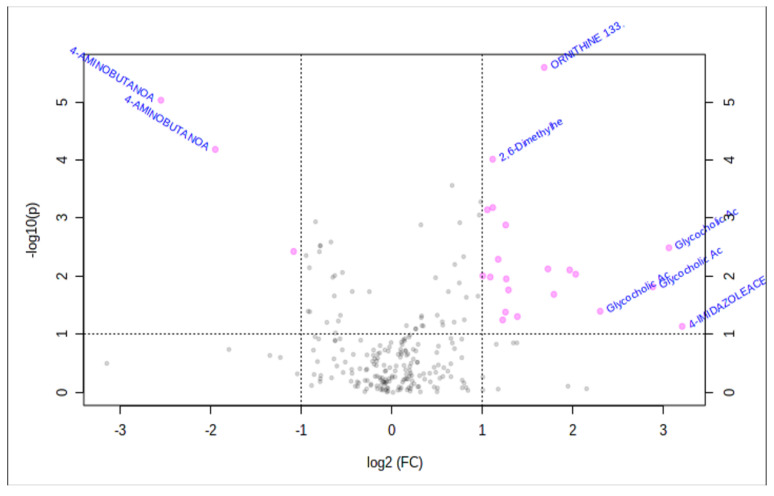
Volcano plot. Illustrates the scatter of metabolites with increased significance at the extremes of the plot.

Analysis using a volcano plot (Figure 2) demonstrates a few metabolites that are more significantly isolated between the groups, specifically gamma aminobutanoate (GABA). This neurotransmitter correlated with certain microbial taxa, as described previously [20].

### 2.3. Metabolomics-Comparison of Randomized Groups

**Figure 3:**
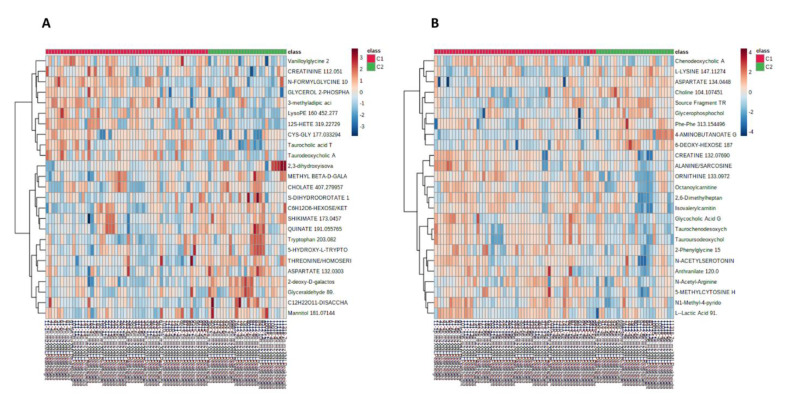
Heat map. Color gradients depicting increased (red) versus decreased (blue) concentrations. (**A**) Top 25 negative ion metabolites (*p* < 0.05). (**B**) Top 25 positive ion metabolites (*p* < 0.05).

As differences were observed in the PLS–DA and volcano plots, individual metabolites were compared. Here we will focus on several differences between within the infants randomized to receive or not receive antibiotics (C1 vs. C2). The top 25 positive ion and 25 negative ion metabolites isolated within these samples are depicted via a heat map in Figure 3. It is apparent on these heat maps that the antibiotic group (red) and the no antibiotic group (green) not only have differences between them as denoted by the darkening red or blue colors, but also appear to encompass categories of metabolites that will be discussed below.

**Figure 4:**
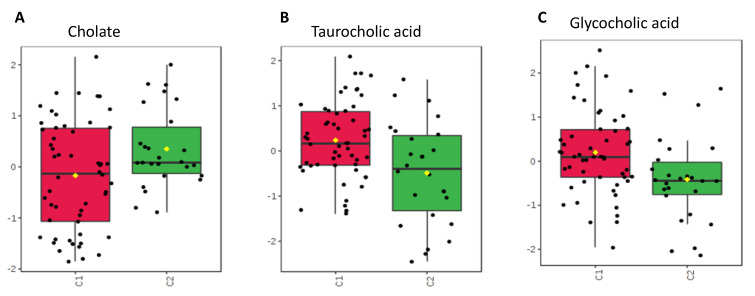
Concentrations compared between groups C1 and C2. (**A**) Primary bile acid, cholate, with unchanged level despite antibiotic administration (*p* > 0.05). (**B**,**C**) Conjugated bile acids taurocholic acid and glycocholic acid. Both with increased expression in the group given antibiotics (*p* = 0.043 for taurocholic acid, *p* = 0.047 for glycocholic acid).

**Figure 5:**
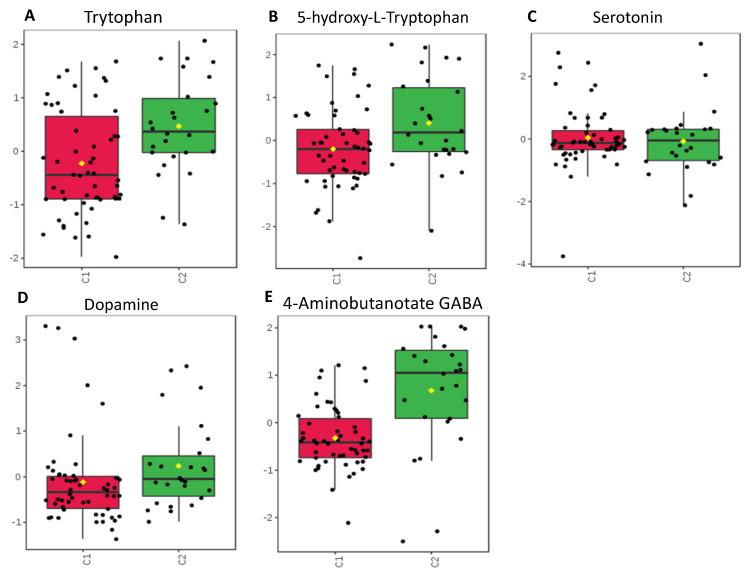
Neurotransmitters. Fecal concentrations compared between groups C1 and C2. (**A**–**D**) Neurotransmitters falling within the serotonin pathway: tryptophan (*p* = 0.047), 5-HT (*p* = 0.17), serotonin (*p* = 0.82), dopamine (*p* = 0.40). (**E**) GABA (*p* = 0.001), a neurotransmitter which is produced in high quantities within enterocytes.

**Figure 6:**
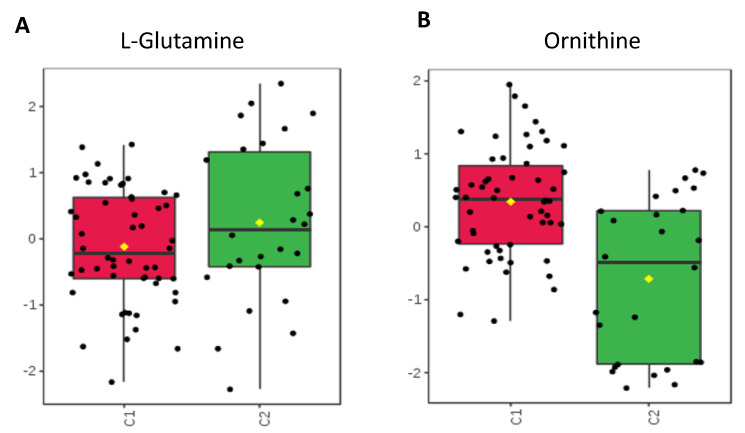
Amino Acids. Fecal concentrations compared between groups C1 and C2. (**A**) Glutamine, with a trend of decreased concentration in the group exposed to antibiotics (*p* > 0.05). (**B**) Ornithine, with increased concentration in the group exposed to antibiotics (*p* = 0.001).

**Figure 7:**
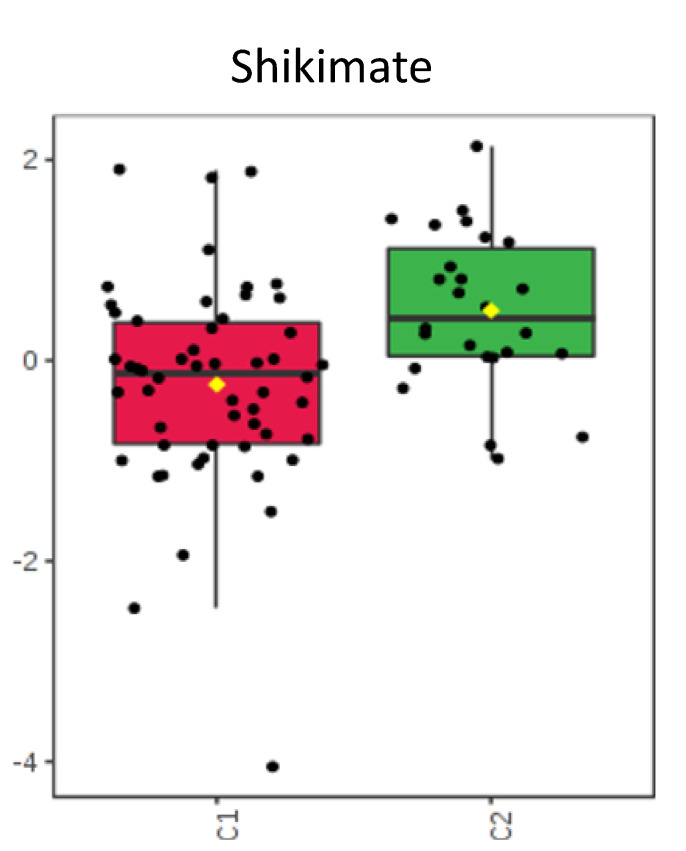
Concentrations compared between groups C1 and C2. Shikimate, bacterial metabolite important within the shikimate-folate pathway (*p* = 0.043).

Individual metabolites were compared using T-tests. The metabolites exhibiting significant (*p* < 0.05) differences or intriguing trends are described in Table 2, where we also provide their function and group them into functional guilds. Figure 4, Figure 5, Figure 6 and Figure 7 show these groupings. The values in Figure 4, Figure 5, Figure 6 and Figure 7 are sum normalized, log transformed and autoscaled (mean centered and divided by the standard deviation of each variable). The *y*-axis represents those normalizations.

### 2.4. Bile Acid

Primary bile acids, such as cholate, and conjugated bile acids, such as taurocholic acid and glycocholic acid, figured more prominently within stool collected from the antibiotic group. No significant difference was seen in cholate level between the groups, while both taurocholic acid (*p* = 0.043) and glycocholic acid (*p* = 0.047) were higher in antibiotic group vs. no antibiotic group.

### 2.5. Neurotransmitters

Neurotransmitters with significant physiologic activity within the gastrointestinal tract also varied between these groups. GABA demonstrated lower concentrations in fecal samples from the antibiotic group (*p* = 0.001). Slight differences were also noted within the serotonin pathway, with the antibiotic group having a decreased fecal tryptophan (*p* = 0.047), while seeming to maintain concentrations of 5-hydroxytryptamine (5-HT) (*p* = 0.17), serotonin (*p* = 0.82), and dopamine (*p* = 0.40).

### 2.6. Amino Acids Important to Intestinal Metabolism

Certain amino acids are highly used within the gastrointestinal tract both within microbes and enterocytes, some of which are involved with intestinal mucosal permeability. Of those isolated within these stool samples, ornithine had a lower fecal concentration within the no antibiotic group (*p* = 0.001).

### 2.7. Shikimate-Folic Acid Metabolism.

Shikimate, used by bacteria in the production of folates and aromatic amino acids, was noted in higher concentrations within the no antibiotic group compared to the antibiotic group (*p* = 0.043). As humans are not able to use or process these metabolites, alterations in their concentration would result from exposure or changes within the gastrointestinal environment.

## 3. Discussion

Antibiotic administration within the NICU is a common practice given concerns for sepsis around the time of delivery and few clinically reliable indicators of neonatal infection. Exposure to antibiotics early in life alters the developing microbiome thereby affecting the metabolome. The gastrointestinal tract plays significant roles in terms of nutrition, immunity, and even neurotransmitter production. Alterations in the microbiome and metabolome using antibiotics could then significantly impair or alter these functions and predispose those babies to future complications. Studies analyzing fecal metabolites published so far have been non-randomized, observational, or retrospective. This study is prospective and randomized, aiming to identify and evaluate metabolites within the stool of preterm neonates specifically related to early antibiotic administration.

Of the isolated metabolites compared between the antibiotic and no antibiotic groups, some show more significant differences and warrant further consideration. These are categorized as follows:

### 3.1. Bile Acids

Some of the more prominently portrayed and variant metabolites are bile acids (Figure 4). The neonates receiving antibiotics showed a higher expression of conjugated bile acids (glycocholic acid and taurocholic acid). These are typically reabsorbed within distal portions of the gastrointestinal tract [23], and this increase may be secondary to decreased absorption into intestinal cells. Some intestinal microbes, such as *Bifidobacteriae*, are also capable of deconjugating these bile acids back into primary acids [24,25], and Russell et al. noted a negative correlation between the abundance of *Bifidobacteriae* and the conjugated bile acids between neonates given antibiotics and those without antibiotics [20]. As antibiotic administration changes the intestinal microbiome, bacteria capable of converting these conjugated bile acids may be significantly reduced or completely gone. This then would lead to increased excretion of glycocholic acid and taurocholic acid and could also explain the diminished expression of cholate in the antibiotic group. Bile acids are important for lipid absorption within the intestines [26], so changes seen between these groups could signify abnormalities of nutrient exchange with implications for nutrition and growth of these neonates.

### 3.2. Neurotransmitters

Tryptophan, serotonin, dopamine, and GABA are all neurotransmitters that are found in high quantities within the gastrointestinal tract. Our results show alterations in fecal expression of these neurotransmitters between groups randomized to receive and to not receive antibiotics (Figure 5). The lower concentration of tryptophan is noted within the group receiving antibiotics, while serotonin appears unchanged. Tryptophan is an essential amino acid, and as such, its presence within the gut is modulated by diet [27]. The gut is also the main source of serotonin synthesis, with over 90% of serotonin being produced by enterochromaffin cells, mucosal mast cells, and myenteric cells [28]. Some microbes, such as Pseudomonas, are capable of converting tryptophan into serotonin for their own metabolism [27]. If antibiotics allow colonies of such microbes to grow, fecal tryptophan levels would diminish, while fecal serotonin would remain stable. The decreased availability of tryptophan to enterocytes may subsequently decrease the amount of serotonin available for systemic distribution. Conversely, if antibiotics decrease the variety of microbes within the gastrointestinal tract, the enterocytes may have increased access to and use of tryptophan to further process to serotonin. Serotonin and dopamine play an important role in gastrointestinal secretion, motility and intestinal blood circulation [29,30], with abnormal levels being implicated in diseases such as irritable bowel syndrome and secretory diarrhea [29,31]. Fecal serotonin was unchanged between the randomized groups which maybe secondary to antibiotics decreasing bacterial levels allowing for formation of serotonin producing microbes or for increased use by enterocytes.

GABA was also found in lower concentrations in the group randomized to receive antibiotics. GABA is produced within the myenteric plexus and mucosal endocrine cells and plays a role in contraction and relaxation of enteric muscles. GABA may also play a role in gastrointestinal immunity [32]. As a neurotransmitter and part of the gut-brain axis, GABA is important for neurodevelopment [33]. Russell et al. showed that GABA concentrations were negatively impacted by the administration of antibiotics, at least partly due to decreased colonization with *Veillonella* [20]. *Lactobacillus* and *Bifidobacteria* have also been shown to produce GABA [34]. Changes in the microbiota from antibiotics could lead to significant alterations in GABA producing bacteria which may have contributed to our results. Given our developing understanding of the gut-brain axis and GABA’s role in neurodevelopment and immunity, decreased levels of GABA in stool may imply a larger effect on neonatal development.

### 3.3. Amino Acids Critical to Intestinal Metabolism

Ornithine and glutamine are amino acids highly used within the healthy gastrointestinal tract. Glutamine provides an energy source for enterocytes and aids with protein synthesis as well as differentiation of enterocytes [35]. Models of sepsis and gut inflammation have shown decreased uptake of glutamine by enterocytes [35]. Supplementation with glutamine has been studied in prevention of NEC in preterm infants, as it has been found to decrease the possibility of bacterial translocation across enterocytes [36]. The decreased concentration in the antibiotic group may be secondary to increased use in maintenance of the mucosal barrier and in further proliferation of enterocytes (Figure 6). This response may be from a reaction within the enterocytes due to microbial changes caused by the short course of antibiotics and may demonstrate degradation of metabolic and immune function of these enterocytes. Similarly, enterocytes actively transport and use ornithine as an important energy source and it is important for appropriate growth [37] as it can stimulate the release of growth hormone [38]. Elevated levels in the stool of the antibiotic group suggest that absorption into enterocytes likely is impaired. This disruption could impact the overall growth and subsequent development of a neonate.

### 3.4. Shikimate

Shikimate is a metabolite whose presence within the fecal metabolome is completely dependent on production by intestinal microbes [39]. It is noted within both groups but has a larger concentration within the no antibiotic group (Figure 7). Alterations in its expression are associated with antibiotic use and suggest that even a short course of antibiotics can produce a significant change in these important metabolites.

### 3.5. Strengths and Limitations

The strengths in this study include its prospective and randomized design, which allows for better comparison of groups and attempts to prevent bias. The sample size in this study was limited due to financial constraints related to metabolome testing. This limited both the number of patients eligible for analysis as well as the number of stool samples tested per patient. A larger sample size would allow for a more comprehensive analysis of the metabolome of these preterm patients throughout their hospitalization.

Urine remains a source of possible contamination, as we could not guarantee that stool was separate from urine during sample collection. Some metabolites isolated were not identified within the national database and were not mentioned; however, if identified as the national database grows, they could hold significance.

## 4. Methods

### 4.1. Clinical Trial

**Figure 8:**
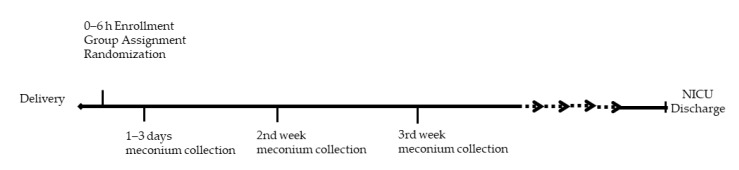
Study Collection Timeline.

**Figure 9:**
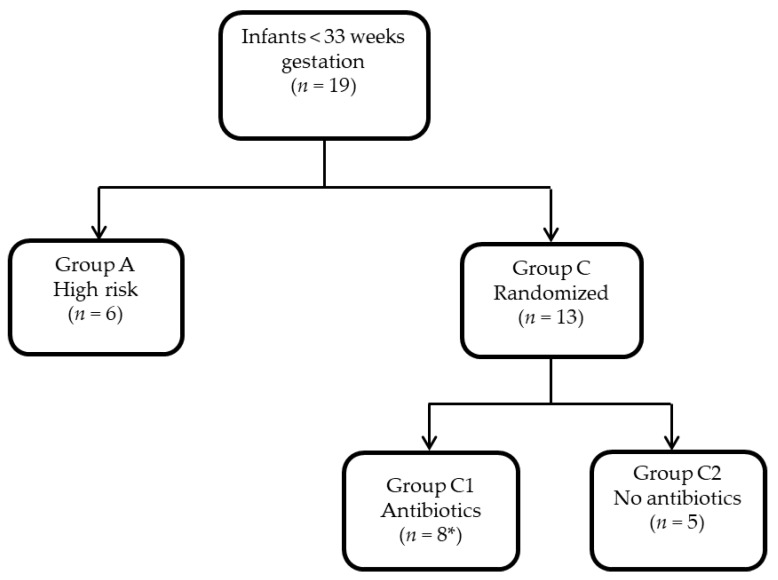
Group schematic. * Represents the total after 3 were bailed from Group C2.

The REASON study was conducted from January 2017–January 2019 at the University of Florida after approval by the institutional review board (IRB201501045). A detailed description of the study design including enrollment, group selection, randomization, and collection of clinical samples and data has been previously described [19]. Briefly, 98 infants < 33 weeks’ gestation were enrolled and placed into one of three groups according to previously described criteria: group A neonates—high risk for infection with indication for antibiotics; group B—asymptomatic babies with low risk for infection without indication for antibiotics; and group C—symptomatic babies with low risk for infection, eligible for randomization to antibiotics (C1-abx) or no antibiotics (C2-no abx). Infants in C1-abx were placed on antibiotics for 48 h after birth. Infants could be continued on antibiotics longer than 48 h in group C1-abx or started on antibiotics within 48 h after birth in group C2-no abx based on the medical team’s judgement. All infants who received antibiotics were initially placed on ampicillin and gentamicin, with the ability to broaden if deemed medically necessary by the providers. Informed consent was obtained prior to enrollment and randomization. Because of financial limitations, only the first 19 patients, including group A (*n* = 6, with 43 weekly samples), group C1 (*n* = 8, with 54 weekly samples) and group C2 (*n* = 5, with 26 weekly samples) from the REASON trial [19] underwent fecal metabolite analysis and are presented in this paper. The study collection timeline and schematic of patient groups are depicted in Figure 8 and Figure 9 respectively.

### 4.2. Stool Sample Collection

Weekly fecal samples, including first meconium when possible, were collected from the patient diaper into a sterile container and stored at −80 °C. For protein normalization, stool samples were first suspended in 5 mM Ammonium Acetate (400 μL) and then homogenized using a cell disrupter (30 s, 3 times). Protein concentrations were determined with a Qubit (ThermoScientific, Waltham, MA, USA). All samples were normalized to a protein concentration of 500 µg/mL at 25 µL. The normalized samples were spiked with 5 µL of internal standards containing Creatine-D3, L-Leucine-D10, L-Tryptophan-2,3,3-D3, L-Tyrosine Ring-^13^C6, L-Leucine ^13^C6, L-Phenylalanine Ring-^13^C6, N-BOC-L-tert-Leucine, N-BOC-L-Aspartic Acid, Propionic Acid ^13^C3, Succinic Acid-2,2,3,3-D4, Salicylic Acid-D4, and Caffeine-D3 (1-methyl-d3). Metabolites were extracted by protein precipitation with the addition of 200 µL of 8:1:1 Acetonitrile: Methanol: Acetone, followed by vortex mixing. The samples were then incubated at 4 °C to allow additional protein precipitation, and finally centrifuged 20,000× *g* to pellet the protein. Only 190 µL of the supernatant was transferred and dried down using nitrogen. Samples were reconstituted with 25 µL of 0.1% formic acid containing injection standards (BOC-L-Tyrosine, BOC-L-Tryptophan, BOC-D-Phenylalanine), vortex mixed, and incubated at 4 °C for 10–15 min, followed by centrifugation at 20,000× *g*. The reconstituted samples were then transferred into LC-vials.

### 4.3. Metabolomic Profiling

Global metabolomics analysis was conducted using a Thermo Q-Exactive Orbitrap mass spectrometer with Dionex ultra high performance liquid chromatography (UHPLC) and autosampler [40] (ThermoFisherScientific). Samples were injected in both positive and negative heated electrospray ionization as separate analyses. The mass resolution was 35,000. Separation was achieved on an ACE C18-pfp column (100 × 2.1 mm, 2 µm, ACE chromatography, Aberdeen, Scotland). Mobile phase A was 0.1% formic acid in water and mobile phase B was unmodified acetonitrile. A flow rate of 350 µL/min was used with a column temperature of 25 °C. The injection volume was 4 µL for negative ions and 2 µL for positive ions.

Data from positive and negative ions modes were processed separately. Native files (.RAW) were converted to an open-format (mzXML) using MSConvert (Proteowizard). The open source software MZmine was used for feature identification including deisotoping, alignment and gap filling. Features were then matched to an internal compound retention time database to identify metabolites [41]. Known adducts and complexes were identified by mass accuracy and retention time and removed from the data set prior to statistical analysis.

### 4.4. Statistical Analysis

Metaboanalyst 3.0 was used for all statistical analysis, including partial least squares discriminant analysis (PLS-DA), volcano plot, ANOVA, *t*-tests, heatmap analysis and individual comparison of the metabolites that exhibited the highest significance as determined by *p*-value less than or equal to 0.05 [42]. Where appropriate, false discovery rate (FDR)-corrected *p*-values were used. The heatmap was generated from the top 25 metabolites as determined by *p*-values.

## 5. Limitations and Future Directions

Despite a limited sample size, several significant associations were found in fecal metabolites based on antibiotic use. In future studies, a larger sample size will allow for analysis of these metabolites throughout the course of each neonate’s hospitalization and in response to individual antibiotic courses. As the samples in this study were obtained until funding was depleted, a larger sample allowance would provide data to delineate changes based on diet, change in diet, and age after delivery.

Across these samples, we showed a variety of isolated metabolites, although only a small proportion of these are currently identifiable. As more information is collected through all research involving metabolites, the national database that we use for purposes of identification will grow and more of these unknowns will be able to be categorized and used for theories of importance. Despite these limitations, the antibiotic associated alterations in metabolites seen during the critical developmental window of preterm infants prompts the need for a more in-depth study, the results of which could be considered when the decision is made to routinely provide antibiotics without clear evidence of infection.

## Figures and Tables

**Table 1:** Patient information.

Group, Patient	Gestational Age (wks)	Antibiotics	Feeding Type *	Number of Stool Samples
**A**	Mean 29.5			43
**5**	28	Ampicillin, Gentamicin, Piperacillin-tazobactam, Vancomycin	DBM, MBM	9
**6**	28	Ampicillin, Azithromycin, Fluconazole, Gentamicin, Metronidazole, Piperacillin-tazobactam, Vancomycin	DBM, MBM, formula	12
**14**	32	Ampicillin, Gentamicin	formula	5
**17**	31	Ampicillin, Gentamicin	DBM, MBM	7
**19**	26	Ampicillin, Cefotaxime, Fluconazole, Gentamicin, Metronidazole, Piperacillin-tazobactam, Vancomycin	DBM, MBM, formula	9
**20**	32	Ampicillin, Ceftazidime, Cefotaxime, Fluconazole, Gentamicin, Metronidazole, Oxacillin, Piperacillin-tazobactam, Rifampin, Vancomycin	DBM, formula	1
**C1**	Mean 27			54
**1**	26	Ampicillin, Gentamicin, Vancomycin	DBM, MBM, formula	14
**2**	28	Ampicillin, Azithromycin Fluconazole, Gentamicin, Metronidazole, Piperacillin-tazobactam, Vancomycin	MBM, formula	13
**3**	25	Azithromycin, Piperacillin-tazobactam, Fluconazole	DBM, MBM, formula	11
**7**	28	Ampicillin, Fluconazole, Gentamicin, Piperacillin-tazobactam	MBM	1
**10**	29	Ampicillin, Gentamicin	NPO	4
**11**	24	Ampicillin, Gentamicin, Piperacillin-tazobactam, Vancomycin	NPO	2
**13**	28	Ampicillin, Gentamicin	NPO	3
**15**	29	Ampicillin, Gentamicin	DBM, MBM, formula	6
**C2**	Mean 28			26
**4**	29	No	DBM, MBM, formula	7
**8**	32	No	MBM, formula	2
**12**	27	No	DBM, MBM, formula	10
**16**	23	No	NPO	1
**18**	29	No	DBM, EBM	6

DBM = donor breast milk; MBM = mother’s breast milk; NPO = nil per os. * Feeding type is listed for all types of feeds during stool sample collection.

**Table 2:** Classification, summary, and interpretation of select fecal metabolites.

Category	Metabolites	*p*-Value	Group Effect	Significance
Bile Acids	Primary Bile Acids: Cholate Conjugated Bile Acids Taurocholic acid Glycocholic acid	>0.05 0.043 0.047	- C1 = C2 C1 > C2	Increased presence of conjugated bile acids in the stool may be due to diminished absorption to enterocytes and decreased deconjugation by enteral microbes.
Neurotransmitters	Serotonin Pathway: Tryptophan 5-HT Serotonin Dopamine GABA	0.047 0.17 0.82 0.4 0.001	C1 < C2 C1 < C2 C1 = C2 C1 < C2 C1 < C2	Decreased tryptophan in C1 but with equivalent serotonin is suggestive that enterocytes and gut microbes have altered their use of tryptophan or have a level of impairment of this pathway. May indicate decreased production or release from enterocytes into the GI tract or perturbations of GABA producing microbes. Overall changes suggest alteration of gut-brain axis and possible implications for neurodevelopment.
Amino Acids	Glutamine Ornithine	>0.05 0.001	C1 < C2 C1 > C2	Suggestive of increased transport into or use by enterocytes. Suggestive of impaired uptake by enterocytes. Implies possibility of growth impairment.
Microbial	Shikimate	0.043	C1 < C2	Present only within bacteria. Diminished in C1 due to antibiotics.

## References

[1] Clark R.H., Bloom B.T., Spitzer A.R., Gerstmann D.R. (2006). Reported medication use in the neonatal intensive care unit: Data from a large national data set. Pediatrics.

[2] Klingenberg C., Kornelisse R.F., Buonocore G., Maier R.F., Stocker M. (2018). Culture-Negative Early-Onset Neonatal Sepsis—At the Crossroad Between Efficient Sepsis Care and Antimicrobial Stewardship. Front. Pediatr..

[3] Hsieh E.M., Hornik C.P., Clark R.H., Laughon M.M., Benjamin D.K., Smith P.B. (2014). Medication use in the neonatal intensive care unit. Am. J. Perinatol..

[4] Cotten C.M., Taylor S., Stoll B., Goldberg R.N., Hansen N.I., Sánchez P.J., Ambalavanan N., Benjamin D.K. (2009). Prolonged duration of initial empirical antibiotic treatment is associated with increased rates of necrotizing enterocolitis and death for extremely low birth weight infants. Pediatrics.

[5] Alexander V.N., Northrup V., Bizzarro M.J. (2011). Antibiotic exposure in the newborn intensive care unit and the risk of necrotizing enterocolitis. J. Pediatr..

[6] Kuppala V.S., Meinzen-Derr J., Morrow A.L., Schibler K.R. (2011). Prolonged initial empirical antibiotic treatment is associated with adverse outcomes in premature infants. J. Pediatr..

[7] Lee-Sarwar K.A., Lasky-Su J., Kelly R.S., Litonjua A.A., Weiss S.T. (2020). Metabolome—Microbiome Crosstalk and Human Disease. Metabolites.

[8] Hanson M.A., Gluckman P.D. (2014). Early developmental conditioning of later health and disease: Physiology or pathophysiology?. Physiol. Rev..

[9] Indrio F., Martini S., Francavilla R., Corvaglia L., Cristofori F., Mastrolia S.A., Neu J., Rautava S., Russo Spena G., Raimondi F. (2017). Epigenetic Matters: The Link between Early Nutrition, Microbiome, and Long-term Health Development. Front. Pediatr..

[10] Antunes L.C., Han J., Ferreira R.B., Lolić P., Borchers C.H., Finlay B.B. (2011). Effect of antibiotic treatment on the intestinal metabolome. Antimicrob. Agents Chemother..

[11] Antharam V.C., McEwen D.C., Garrett T.J., Dossey A.T., Li E.C., Kozlov A.N., Mesbah Z., Wang G.P. (2016). An Integrated Metabolomic and Microbiome Analysis Identified Specific Gut Microbiota Associated with Fecal Cholesterol and Coprostanol in Clostridium difficile Infection. PLoS ONE.

[12] Young V.B., Schmidt T.M. (2004). Antibiotic-associated diarrhea accompanied by large-scale alterations in the composition of the fecal microbiota. J. Clin. Microbiol..

[13] Jump R.L., Polinkovsky A., Hurless K., Sitzlar B., Eckart K., Tomas M., Deshpande A., Nerandzic M.M., Donskey C.J. (2014). Metabolomics analysis identifies intestinal microbiota-derived biomarkers of colonization resistance in clindamycin-treated mice. PLoS ONE.

[14] Zhao Y., Wu J., Li J.V., Zhou N.Y., Tang H., Wang Y. (2013). Gut microbiota composition modifies fecal metabolic profiles in mice. J. Proteome Res..

[15] Langdon A., Crook N., Dantas G. (2016). The effects of antibiotics on the microbiome throughout development and alternative approaches for therapeutic modulation. Genome Med..

[16] Zhu D., Ziao S., Yu J., Ai Q., He Y., Cheng C., Zhang Y., Pan Y. (2017). Effects of One-Week Empirical Antibiotic Therapy on the Early Development of Gut Microbiota and Metabolites in Preterm Infants. Sci. Rep..

[17] Stewart C.J., Skeath T., Nelson A., Fernstad S.J., Marrs E.C., Perry J.D., Cummings S.P., Berrington J.E., Embleton N.D. (2015). Preterm gut microbiota and metabolome following discharge from intensive care. Sci. Rep..

[18] Wandro S., Osborne S., Enriquez C., Bixby C., Arrieta A., Whiteson K. (2018). The Microbiome and Metabolome of Preterm Infant Stool Are Personalized and Not Driven by Health Outcomes, Including Necrotizing Enterocolitis and Late-Onset Sepsis. mSphere.

[19] Ruoss J.L., Bazacliu C., Russell J.T., de la Cruz D., Li N., Gurka M.J., Filipp S.l., Polin R., Triplett E.w., Neu J. (2020). Routine Early Antibiotic use in SymptOmatic preterm Neonates (REASON): A prospective randomized controlled trial. medRxiv.

[20] Russell J.T., Ruoss J.L., de la Cruz D., Li N., Bazacliu C., Patton L., McKinley K.L., Garrett T.J., Polin R.A., Triplett E.W. (2020). Antibiotics may influence gut microbiome signaling to the brain in preterm neonates. bioRxiv.

[21] Ge X., Ding C., Zhao W., Xu L., Tian H., Gong J., Zhu M., Li J., Li N. (2017). Antibiotics-induced depletion of mice microbiota induces changes in host serotonin biosynthesis and intestinal motility. J. Transl. Med..

[22] Behr C., Slopianka M., Haake V., Strauss V., Sperber S., Kamp H., Walk T., Beekmann K., Rietjens I.M.C.M., van Ravenzwaay B. (2019). Analysis of metabolome changes in the bile acid pool in feces and plasma of antibiotic-treated rats. Toxicol. Appl. Pharmacol..

[23] Ma N., Ma X. (2018). Dietary Amino Acids and the Gut-Microbiome-Immune Axis: Physiologic Metabolism and Therapeutic Prospects. Compr. Rev. Food Sci. Food Saf..

[24] Grill J.P., Manginot-Dürr C., Schneider F., Ballongue J. (1995). Bifidobacteria and probiotic effects: Action of Bifidobacterium species on conjugated bile salts. Curr. Microbiol..

[25] Jia W., Xie G. (2018). Bile acid-microbiota crosstalk in gastrointestinal inflammation and carcinogenesis. Nat. Rev. Gastroenterol. Hepatol..

[26] Hofmann A.F. (1999). The continuing importance of bile acids in liver and intestinal disease. Arch. Intern. Med..

[27] Kaur H., Bose C., Mande S.S. (2019). Tryptophan Metabolism by Gut Microbiome and Gut-Brain-Axis: An in silico Analysis. Front. Neurosci..

[28] Yano J.M., Yu K., Donaldson G.P., Shastri G.G., Ann P., Ma L., Nagler C.R., Ismagilov R.F., Mazmanian S.K., Hsiao E.Y. (2015). Indigenous bacteria from the gut microbiota regulate host serotonin biosynthesis. Cell.

[29] Camilleri M. (2009). Serotonin in the gastrointestinal tract. Curr. Opin. Endocrinol. Diabetes Obes..

[30] Shajib M.S., Khan W.I. (2015). The role of serotonin and its receptors in activation of immune responses and inflammation. Acta Physiol..

[31] Eisenhofer G., Aneman A., Friberg P., Hooper D., Fåndriks L., Lonroth H., Hunyady B., Mezey E. (1997). Substantial production of dopamine in the human gastrointestinal tract. J. Clin. Endocrinol. Metab..

[32] Auteri M., Zizzo M.G., Serio R. (2015). GABA and GABA receptors in the gastrointestinal tract: From motility to inflammation. Pharmacol. Res..

[33] Kwon S.H., Scheinost D., Lacadie C., Benjamin J., Myers E.H., Qiu M., Schneider K.C., Rothman D.L., Constable R.T., Ment L.R. (2014). GABA, resting-state connectivity and the developing brain. Neonatology.

[34] Galland L. (2014). The gut microbiome and the brain. J. Med. Food.

[35] Gardiner K.R., Kirk S.J., Rowlands B.J. (1995). Novel substrates to maintain gut integrity. Nutr. Res. Rev..

[36] El-shimi M.S., Awad H.A., Abdelwahed M.A., Mohamed M.H., Khafagy S.M., Saleh G. (2015). Enteral L-Arginine and Glutamine Supplementation for Prevention of NEC in Preterm Neonates. Int. J. Pediatr..

[37] Cynober L. (1994). Can arginine and ornithine support gut functions?. Gut.

[38] Ho Y.Y., Nakato J., Mizushige T., Kanamoto R., Tanida M., Akiduki S., Ohinata K. (2017). l-Ornithine stimulates growth hormone release in a manner dependent on the ghrelin system. Food Funct..

[39] Mir R., Jallu S., Singh T.P. (2015). The shikimate pathway: Review of amino acid sequence, function and three-dimensional structures of the enzymes. Crit. Rev. Microbiol..

[40] Chamberlain C.A., Hatch M., Garrett T.J. (2019). Metabolomic and lipidomic characterization of Oxalobacter formigenes strains HC1 and OxWR by UHPLC-HRMS. Anal. Bioanal. Chem..

[41] Pluskal T., Castillo S., Villar-Briones A., Oresic M. (2010). MZmine 2: Modular framework for processing, visualizing, and analyzing mass spectrometry-based molecular profile data. BMC Bioinform..

[42] Pang Z., Chong J., Li S., Xia J. (2020). MetaboAnalystR 3.0: Toward an Optimized Workflow for Global Metabolomics. Metabolites.

